# Sex Differences in Circadian Dysfunction in the BACHD Mouse Model of Huntington’s Disease

**DOI:** 10.1371/journal.pone.0147583

**Published:** 2016-02-12

**Authors:** Dika A. Kuljis, Laura Gad, Dawn H. Loh, Zoë MacDowell Kaswan, Olivia N. Hitchcock, Cristina A. Ghiani, Christopher S. Colwell

**Affiliations:** 1 Department of Neurobiology, University of California Los Angeles, Los Angeles, California, United States of America; 2 Department of Psychiatry & Biobehavioral Sciences, University of California Los Angeles, Los Angeles, California, United States of America; 3 Department of Pathology and Laboratory Medicine, University of California Los Angeles, Los Angeles, California, United States of America; University of Lübeck, GERMANY

## Abstract

Huntington’s disease (HD) is an autosomal dominant neurodegenerative disorder that affects men and women in equal numbers, but some epidemiological studies indicate there may be sex differences in disease progression. One of the early symptoms of HD is disruptions in the circadian timing system, but it is currently unknown whether sex is a factor in these alterations. Since sex differences in HD could provide important insights to understand cellular and molecular mechanism(s) and designing early intervention strategies, we used the bacterial artificial chromosome transgenic mouse model of HD (BACHD) to examine whether sex differences in circadian behavioral rhythms are detectable in an animal model of the disease. Similar to BACHD males, BACHD females display circadian disruptions at both 3 and 6 months of age; however, deficits to BACHD female mouse activity levels, rhythm precision, and behavioral fragmentation are either delayed or less severe relative to males. These sex differences are associated with a smaller suprachiasmatic nucleus (SCN) in BACHD male mice at age of symptom onset (3 months), but are not associated with sex-specific differences in SCN daytime electrical activity deficits, or peptide expression (arginine vasopressin, vasoactive intestinal peptide) within the SCN. Notably, BACHD females exhibited delayed motor coordination deficits, as measured using rotarod and challenge beam. These findings suggest a sex specific factor plays a role both in non-motor and motor symptom progression for the BACHD mouse.

## Introduction

Huntington’s disease (HD) is caused by a toxic gain-of-function mutation in the huntingtin gene (*HTT*) that induces progressive neurodegeneration and dysfunction resulting in motor (chorea and dystonia), psychiatric (cognitive changes and depression), and other non-motor (sleep, hormonal, and metabolic) symptoms [1]. Typically, the HD diagnosis is made in the 4^th^ or 5^th^ decade of life when, following a threshold of neurodegeneration in brain regions regulating movement, characteristic motor symptoms emerge [1,2]. The HD process is believed to be well-underway by this point, as prodromal symptoms including disrupted sleep and altered cognition, mood, and hormonal profiles commonly occur years beforehand [3–14]. Even in otherwise healthy individuals, a disrupted sleep/wake cycle can cause a cluster of health problems (cognitive dysfunction, cardiovascular disease, metabolic dysfunction, affective disorder, gastro-intestinal disturbance, and impaired immune system function) [15–23], all of which are also experienced by HD patients [3–5,8,9,24–29]. This overlap raises the possibility that disrupted circadian system is not only a symptom of HD, but also that this early disruption triggers a host of health problems in gene carriers that feed forward into the HD pathology and accelerate disease progression.

HD patients exhibit alterations in the timing of daily rhythms in activity, sleep, and melatonin rhythms [8,24,30–32] which led us to further investigate the circadian timing system in the BACHD (bacterial artificial chromosome HD) mouse model. Prior work has shown that the BACHD model exhibits classic HD symptoms (progressive motor, cognitive, and affective) [33–35] and a delayed sleep onset phenotype similar to the delayed onset sleep insomnia reported in HD patients [36]. In addition, the central circadian clock (suprachiasmatic nucleus, SCN) of male BACHD mice loses the ability to generate rhythmic electrical output, and this correlates with reductions in behavioral and physiological rhythm amplitude [36]. Therefore, we consider the BACHD line a useful preclinical model to explore circadian disruption in HD and to evaluate potential therapeutic interventions. While males and females are diagnosed with HD in equal numbers, some epidemiological studies have found sex differences in the age of onset, duration, or severity of the HD symptoms [37–40] while others have not [41, 42]. In the present study, we sought to determine whether there were sex differences in circadian disruption in the BACHD model. New preclinical guidelines call for gender balance in experimental design and thus possible sex differences in behavior and physiology need to be evaluated as research moves forward with the BACHD model.

## Materials and Methods

### Animals and housing

All experimental protocols used in this study were approved by the UCLA Animal Research Committee, and followed guidelines and recommendations for animal use and welfare set by the UCLA Division of Laboratory Animal Medicine and National Institutes of Health. The mouse model of HD (BACHD) we employed for this study, transgenically expresses the full length human mutant huntingtin gene encoding 97 glutamine repeats under the control of endogenous regulatory machinery [33]. In our own breeding facility at UCLA, female BACHD dams, backcrossed on a C57BL/6J background (minimum 12 generations), were bred with C57BL/6J (WT) males from The Jackson Laboratory (Bar Harbor, Maine) to obtain male and female offspring, either WT or heterozygous for the BACHD transgene. Genotyping was performed at 15 days of age by tail snips, and after weaning naïve littermates were group housed by sex, until otherwise noted. Experiments testing behavioral rhythms, motor function, and body weight used WT and BACHD mice from 3–7 months of age (n = 8, per group), while SCN physiology and SCN anatomy focused on 3-month-old animals (n = 3–7, per group). All animals were housed in sound proof, humidity controlled chambers with controlled lighting conditions, using a 12 hour light, 12 hour dark cycle (12:12 LD, intensity 300 lux), unless otherwise noted.

### Rhythms in locomotor activity

Methods employed were as previously reported [36,43]. Mice were individually housed in cages equipped with running wheels, and their voluntary wheel-running activity was recorded as revolutions (rev) per 3 minute intervals using a data acquisition system obtained from Mini Mitter Co. (Bend, OR). Diurnal rhythms in running wheel activity were assessed over 14 days while animals were exposed to a 12:12 LD cycle, and circadian rhythms were assessed in constant darkness (DD) over the subsequent 10 days. To determine the effects of a phase delaying light treatment, a pulse of white light (100 lux, 10 min) was applied at circadian time (CT) 16 after 10–14 days in DD. The resulting phase shift was calculated from the 10 days subsequent to the light-pulse as previously described [43]. As is the convention, CT 12 was defined as the time of activity onset. All handling of mice in DD was performed with the aid of night vision goggles (FJW Industries, Palantine, IL).

Analysis of locomotor activity rhythms was as described previously [43, 44]. Briefly, we determined the period (hr) and power (%V, rhythm strength) by χ^2^ periodogram analysis. Periodogram-derived period estimates were confirmed using the slope of an eye-fitted line through behavioral onsets. Alpha was defined as the duration of the main activity bout from 10 days of activity, corrected for free-running period in DD. Fragmentation (bouts/day) and precision of day-to-day activity onset were determined using Clocklab (Actimetrics, Wilmette, IL). Fragmentation was defined by bouts/day, where a bout was determined as 21 consecutive minutes of activity (maxgap setting of 21 min). Imprecision was determined by calculating the inverse of daily variation in onset from a best-fit regression line drawn across 10 days of activity. DD imprecision was corrected for free-running period. Phase shift magnitude following the phase-delaying light treatment was determined by measuring the phase difference between best-fit regression lines drawn through the 10 days preceding and 10 days subsequent to the light treatment. Investigators masked as to the experimental group made measurements, and reported values are the average of two independent determinations.

Statistically significant effects of genotype, sex, and age on activity rhythm parameters were tested using Three-Way ANOVA, with *P* < 0.05. When main or interaction effects were identified, significant genotype differences within sex/age, significant sex differences within genotype/age, and significant age differences within genotype/sex were identified post-hoc using the Holm-Sidak method for multiple pairwise comparisons, with *P* < 0.05.

### Electrophysiology

Methods for electrophysiology recordings were similar to those previously reported [43]. Animals were anesthetized with isofluorane before decapitation and brain removal at zeitgeber time (ZT) 2 for daytime recordings (projected ZT 4–6). By convention, ZT 0 is defined as the time of lights ON. Brains were chilled in ice-cold slice solution (in mM: 26 NaHCO_3_, 1.25 NaH_2_PO_4_, 10 glucose, 125 NaCl, 2 KCl, 1 MgCl_2_, 1 CaCl_2_; Sigma-Aldrich) for 5 minutes before trimming and slicing using the Leica VT1200S vibrotome (Nussloch, Germany). Two to three coronal slices (250 μm) containing the SCN were transferred into ice-cold artificial cerebrospinal fluid (ACSF) solution (in mM: 26 NaHCO_3_, 1.25 NaH_2_PO_4_, 10 glucose, 125 NaCl, 3 KCl, 2 MgCl_2_, 2 CaCl_2_; Sigma-Aldrich), then incubated at 32°C for 30 minutes before room temperature incubation for one hour. Slices were then placed into a recording chamber (PH-1, Warner Instruments, Hamden, CT) attached to the stage of a fixed upright DIC microscope (Olympus, Tokyo, Japan) and superfused continuously (2 ml/min) with room temperature ACSF. All solutions were adjusted for pH (7.20–7.40) and osmolarity (290–310) and aerated continually with 95% O_2_/ 5% CO_2_ for at least 15 minutes before use. Multiple slices containing the SCN were collected, but only mid-most SCN slices were used for recording because at this rostro-caudal level subnuclear populations are relatively anatomically discrete [45]. Mid-SCN was identified based on the morphology of the third-ventricle and optic chiasm, as well as SCN cell density. Recordings were made from neurons in the dorsal SCN region. These neurons typically express AVP, have robust rhythms in electrical activity in the absence of input and were classified by their location just dorsal to the tip of the third ventricle within the mid-SCN slice.

Electrode micropipettes (3–7 MΩ) were used for whole-cell patch clamp recordings. They were pulled from glass capillaries (WPI, Sarasota, FL) using a multistage puller (Sutter P-97, Novato, CA) and filled with standard internal solution (in mM: 112.5 K-gluconate, 4 NaCl, 17.5 KCl, 0.5 CaCl2, 1 MgCl_2_, 5 MgATP, 1 EGTA, 10 HEPES, 1 GTP, 0.1 Leupeptin, 10 Phosphocreatine; pH adjust to 7.2 using KOH, osmolarity adjusted to 290 using sucrose; Sigma-Aldrich). Single-cell recordings were made using the Axopatch 200B amplifier (Molecular Devices, Sunnyvale, CA) and monitored on-line with pCLAMP (Ver. 10, Molecular Devices). The amplifier’s voltage-offset was used to cancel junction potentials between the micropipette’s internal solution and the extracellular solution (ACSF). Cells were approached in voltage-clamp mode (0 mV holding) using slight positive-pressure, then by switching to negative-pressure and gradually lowering the holding potential to -70 mV, a high resistance seal (2–10 GΩ) was formed. A second pulse of negative pressure was used to break the membrane and enter whole-cell mode. In voltage-clamp mode, membrane holding current, cell capacitance, and access resistance were tested using a 5 mV step applied at 5 Hz from the -70 mV holding potential. Only cells with holding current between 0 and -20 pA, capacitance less than 20 pF, and access resistance less than 60 pA (typically 10 to 40 MΩ) were used. These parameters were monitored during the course of the experiment, and if they changed significantly that cell’s data were not included in analysis.

Following the formation of a high-resistance seal and going whole-cell, the amplifier mode was switched from voltage- to current-clamp, and baseline spontaneous firing rate (SFR) was recorded during the subsequent minute. No current injection was applied during SFR recordings. SFR was calculated as the total number of action potentials recorded during 1 minute. Daytime SFRs for male and female, WT and BACHD mice were determined by averaging data from between 10 and 20 neurons collected from a minimum of 3 animals per group. Action potential (AP) properties were analyzed using Clampfit software’s event detection feature (Ver. 10.4, Molecular Devices).

Resting membrane properties were examined in separate slices from SFR experiments. Before resting membrane properties were measured, slices were treated for at least 2 minutes with TTX (1 μM; Tocris Bioscience, Minneapolis, MN) and gabazine (10 μM) to block action potentials and GABAergic synaptic potentials. Multiple neuronal recordings were attempted per slice, so between recordings drugs were washed off for 2–5 minutes (long enough to partially wash-off TTX and gabazine from voltage-gated sodium and GABA channels), and the partial restoration of electrical membrane events was used to discriminate electrically active neurons from non-electrically active cells (glia), both of which are present in the SCN. Similar to SFR experiments, after forming a high resistance seal and going into whole-cell mode in voltage-clamp (-70 mV), the amplifier was immediately switched to current-clamp mode. Typically, neurons reached a stable membrane potential within the first 10 seconds of the switch. Resting membrane potential (RMP) was recorded over the subsequent 1 to 3 minute period, and calculated as the average membrane potential during that time. Next, the neuron was treated to hyperpolarizing current steps (500 msec., -5 to -25 pA in 5 pA steps) 3 consecutive times. Resulting traces were filtered for electrical interference before analysis (harmonics 1:1, 119 cycles to average, auto-reference frequency). For each current injection step, peak hyperpolarization (using 5 smoothing points) was identified for each of the three replications and averaged. All other parameters (hyperpolarization peak time, area, and slope of the hyperpolarizing membrane response between 10 and 90%) were calculated from an average trace created using the three replications. Cells that were not able to maintain steady membrane potential between current injection treatments were excluded from analysis.

Statistically significant effects of genotype and/or sex on SFR, inter-spike membrane potential, and resting membrane potential were examined using Two-Way ANOVA. When main or interaction effects were identified, significant genotypic differences within sex, and significant sex differences within genotype were identified post-hoc, using the Holm-Sidak method for multiple pairwise comparisons. Statistically significant effects of current injection, sex, and genotype on voltage responses were examined using Three-Way ANOVA. When main or interaction effects were identified, significant genotypic differences within sex and significant sex differences within genotype for each current step were identified post-hoc using Two-Tailed T-Test. For all tests, *P* < 0.05.

### Histological and anatomical analyses of the SCN

Control and mutant mice at 3 months of age were anesthetized with isoflurane (ZT 5–7) and perfused transcardially with 0.9% saline containing heparin (2 units/mL) in 0.1 M phosphate buffer (PB; pH 7.4), followed by 4% paraformaldehyde (0.1 M PB). Brains were post-fixed in 4% PFA at 4°C overnight, and cryopreserved in 20% sucrose (0.1 M PB). Coronal sections (50 μm) were sliced on a cryostat (Leica, Buffalo Grove, IL), collected sequentially and then processed for immunohistochemistry or Nissl staining using cresyl violet, as previously reported [46].

Nissl stained sections were used to estimate the area, height and width of the SCN. Images were acquired on a Zeiss Axioskop with an Axiocam using the AxioVision software (Zeiss, Pleasanton, CA, USA), and measurements (in μm) obtained using this software. Because the borders of the Nissl-defined SCN are somewhat arbitrary, measurements of the left and right SCN were taken by two observers masked as to the animal’s genotype and gender. For each animal, the three measurements were made in consecutive slices of the SCN. Measurements from the 2 most central sections (largest area), the 2 sections anterior and 3 posterior were then summed (for a total of 7 consecutive sections). Both the left and right SCN were measured and no significant differences were found (S1 Table).

VIP and AVP immunostaining was performed as previously reported [46] with minor modifications. Briefly, after blocking (3% normal goat serum, 0.1% Triton X-100 in PBS) free-floating sections (50 μm) were incubated for 48-hour at 4°C with a primary antibody against VIP (rabbit, 1:2000, ImmunoStar; Hudson, WI, USA) or AVP (guinea pig, 1:1000; ImmunoStar, Hudson, WI, USA). Slices were then incubated for 2 hours with the appropriate biotinylated secondary antibody (1:150; Vector Laboratories, Burlingame, CA, USA), followed by incubation with the avidin-biotin complex (Vector Laboratories, Burlingame, CA, USA). Staining was visualized with nickel (II) chloride-enhanced diaminobenzidine (DAB; Sigma) and Nissl-counterstaining performed.

Stereological analysis was performed by a single experimenter (ZAMK) using an AxioImager M2 ApoTome microscope (Zeiss, Pleasanton, CA, USA), equipped with a motorized stage controlled by StereoInvestigator software (MicroBrightField Biosciences, Williston, VT, USA). The area of interest was defined as the entire SCN, and outlined at 10x magnification using anatomical markers and cell density. Due to the SCN’s small area and the low number of VIP^+^ and AVP^+^ neurons in the SCN, stereological parameters were designed to cover the entire area of interest. All immunopositive cell bodies were counted directly at 40x magnification under Köhler illumination.

Statistically significant effects of genotype and sex were examined using Two-Way ANOVA, with *P* < 0.05. Post-hoc examination of genotype and/or sex effects were examined using Mann-Whitney Rank Sum Test or Two-tailed Student’s t Test, with *P* < 0.05. All reported values are Means ± 95% CI.

### Motor Testing

The challenge beam test, a modification of the beam walking test as first described by Fleming and colleagues [47], was performed as previously described [44]. Animals were trained to walk across the beam, from widest to narrowest width, to their home cages during the early night for 2 consecutive days (5 trials/day). On the day of testing, a wire grid (10 x 10 mm spacing) was overlaid onto the beam. Mice were video recorded as they crossed the gridded beam, and videos were scored *post-hoc* by two independent investigators masked as to the genotype and gender for the number of steps taken and step errors made per beam width. We considered an error to be when more than half of the foot in question fell below the grid. Averages of 5 testing trials are reported. Statistically significant effects of sex and age on challenge beam performance for BACHD mice were evaluated using Two-Way ANOVA, with *P* < 0.05. When main or interaction effects were identified, significant sex differences within age, and significant age difference within sex were identified post-hoc using Two-Tailed T-Tests, with *P* < 0.05.

Rotarod testing was performed as previously described [44]. Tests were performed during the early night under dim red light (< 5 lux). Animals were trained for 5 trials (> 1 min rest interval between trials) on an accelerating rotarod apparatus (5 to 38 RPM; Ugo Basile, Varese, Italy). On the next day, mice were placed on the rotarod apparatus and the latency to fall from the rotarod was recorded from 5 trials. An average of the 5 trials is reported. Significant effects of genotype, sex, and/or age on rotarod performance were tested using Three-Way ANOVA, with *P* < 0.05. When main or interaction effects were identified, significant genotypic differences within sex and age, and significant sex differences within genotype and age were identified post-hoc using Two-Tailed T-Tests, with *P* < 0.05

### Body weight

A separate cohort of animals from the same colony was examined weekly for body weight from 2 to 7 months of age. Significant effects of genotype, sex, and/or age on body weight were tested using Three-Way ANOVA, with *P* < 0.05. When main or interaction effects were identified, significant genotypic differences within sex and age, and significant sex differences within genotype and age were identified post-hoc, using the Holm-Sidak method for multiple comparisons, with *P* < 0.05.

## Results

### Delayed deterioration of female BACHD mouse activity rhythm

**Fig 1:**
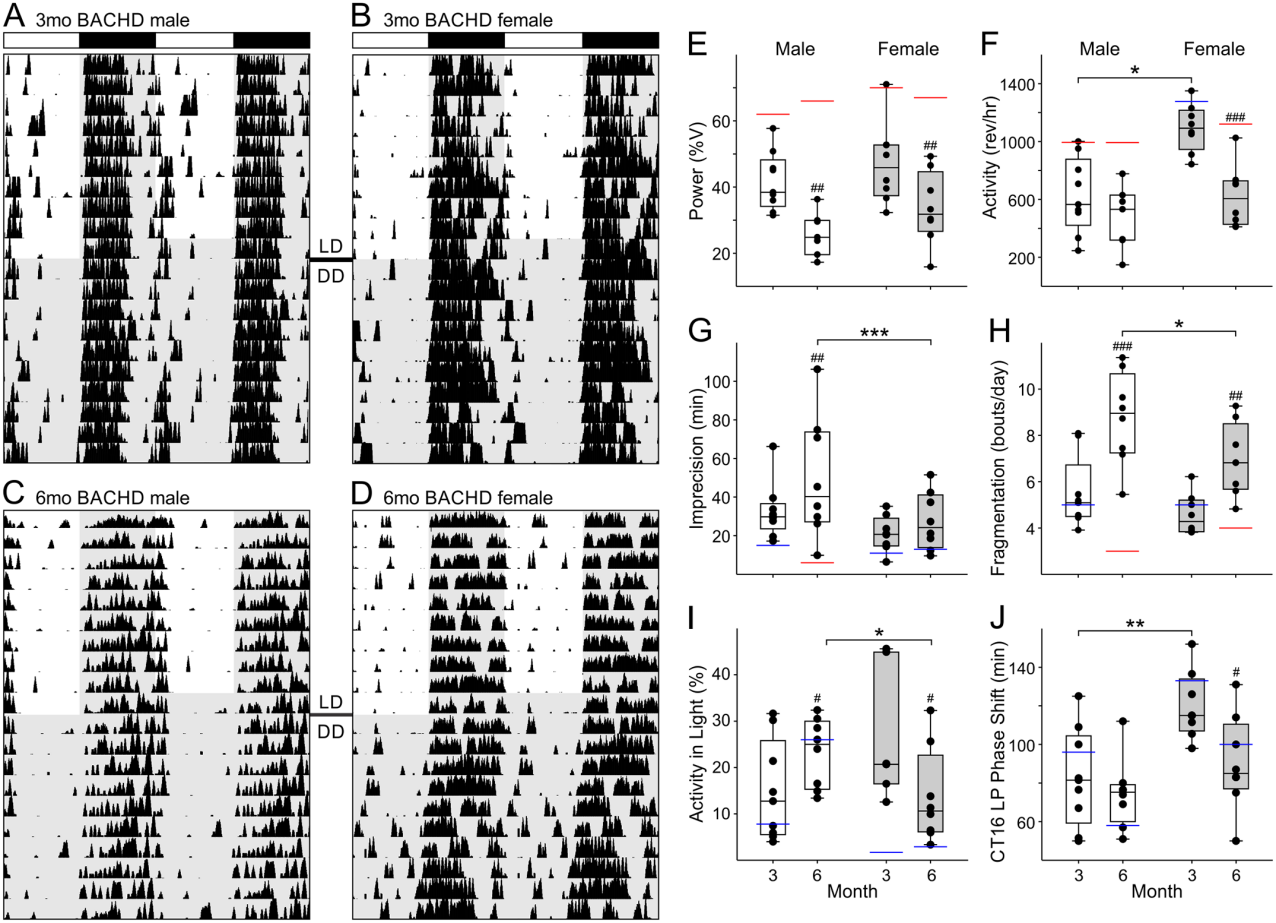
The deterioration of daily and circadian wheel running activity rhythms are delayed in BACHD females. (A-D) Representative double-plotted actograms of BACHD male and female wheel running activity during 10 days in 12:12 LD (300 lux) and DD at 3 and 6 months of age. (E-J) Box plots representing first and third quartile (box), medians (middle line), and data range (whiskers) for male (white boxes) and female (grey boxes) BACHD mouse behavioral rhythm parameters recorded at 3 and 6 months of age in LD (E-H) or DD (I+J). Individual data points (black dots), and WT control median values are superimposed for reference (red lines—statistical significance; blue lines—no statistically significant difference). Three-way ANOVA was used to detect significant effects of genotype, sex, and age on behavioral rhythm parameters (Table 1). When main or interaction effects were identified, significant sex differences within age (*), and significant age differences within sex (^#^), were identified post-hoc, using the Holm-Sidak method for multiple pairwise comparisons, with P < 0.05. Rev/hr refers to wheel revolutions per hour. *P < 0.05; **P < 0.01; ***P < 0.001; ^#^*P* < 0.05; ^##^*P* < 0.01; ^###^*P* < 0.001.

Diurnal and circadian wheel running activity of male and female BACHD mouse was examined at 3 (Fig 1A and 1B), and 6 months of age (Fig 1C and 1D; Tables 1 and 2). Male and female BACHD mice displayed similar age-related decline in the power of their daily rhythms (Fig 1E), but sex differences were detected in a number of other parameters. Firstly, BACHD female activity levels were higher than males in LD and DD at 3 months, before declining to male levels at 6 months (Fig 1F). Secondly, BACHD males experienced age-related decreases in the precision of activity onset so by 6 months their LD activity onsets were significantly less precise than female’s (Fig 1G). Notably, no sex difference in precision was detected when animals were housed in DD and both sexes displayed significantly more day-to-day variation in activity onsets with age. Thirdly, while both BACHD sexes showed age-related increases in activity fragmentation, BACHD males were more severely affected (Fig 1H). Fourthly, BACHD males experienced a progressive increase in light-phase activity so that by 6 months of age they were significantly less nocturnal than BACHD females (Fig 1I). Lastly, although sex differences in photic phase shifting were seen in BACHD mice at 3 months (males exhibited smaller magnitude phase shifts in response to CT16 light-pulse), this sex difference was lost by 6 months of age (Fig 1J; S1 Fig).

**Table 1:** Sex differences in BACHD activity rhythm parameters. Effects of sex and age on BACHD mouse locomotor activity rhythm parameters (DF = 63).

		**WT**		**BACHD**	
	**3 months**	**Male (n = 8)**	**Female (n = 8)**	**Male (n = 8)**	**Female (n = 8)**
**LD**	% V	57.0 ± 6.6	68.1 ± 7.5	41.7 ± 6.8^	47.1 ± 10.3^^^
	Activity (rev/hr)	984 ± 162	1240 ± 213	627 ± 200^^	1093 ± 138***
	% Activity in Light	8.8 ± 4.5	19.8 ± 11.3*	14.8 ± 8.2	23.3 ± 7.4
	Alpha (min)	663 ± 118	690 ± 48.2	727 ± 41.8	712 ± 56.8
	Imprecision (min)	18.6 ± 10.4	11.8 ± 4.7	32.7 ± 11.0	21.0 ± 7.7
	Fragmentation (bouts/day)	5.5 ± 1.2	4.8 ± 0.5	5.5 ± 1.1	4.6 ± 0.7
	LD-DD Phase angle (min)	53.3 ± 48.5	-7.6 ± 19.1*	22.0 ± 23.8	10.5 ± 34.3
**DD**	Period (hr)	23.8 ± 0.2	23.7 ± 0.1	23.9 ± 0.1	23.9 ± .1
	% V	55.5 ± 9.6	63.2 ± 12.4	44.1 ± 7.5	46.5 ± 7.4
	Activity (rev/hr)	1080 ± 75	1250 ± 257	681 ± 187^^^	1190 ± 99***
	Alpha (min)	570 ± 42	639 ± 78*	643 ± 102^	651 ± 64^*
	Imprecision (min)	25.2 ± 14.2	11.7 ± 5.5***	12.6 ± 4.6^	11.8 ± 4.7
	Fragmentation (bouts/day)	4.7 ± 0.8	4.8 ± 0.8	5.7 ± 1.6	5.7 ± 1.1
	CT16 LP (min)	112 ± 32	125 ± 23	82.6 ± 19.4	120 ± 15**
		**WT**		**BACHD**	
	**6 months**	**Male (n = 8)**	**Female (n = 8)**	**Male (n = 8)**	**Female (n = 8)**
**LD**	% V	65.0 ± 8.0	66.4 ± 7.9	25.9 ± 6.1^^^^##^	33.7 ± 9.2^^^^##^
	Activity (rev/hr)	948 ± 177	1070 ± 169	474 ± 201^^^	621 ± 178^^^^###^
	% Activity in Light	2.5 ± 1.5	4.3 ± 2.8^##^	26.0 ± 12.5^^^^#^	13.6 ± 3.6*^#^
	Alpha (min)	524 ± 57.9^###^	692 ± 38.6***	731 ± 92.6^^^	736 ± 40.8
	Imprecision (min)	6.9 ± 1.9	13.9 ± 4.9**	55.5 ± 27.3^^^^##^	27.5 ± 12.5***
	Fragmentation (bouts/day)	4.0 ± 1.0^#^	3.6 ± 0.6	9.2 ± 1.5^^^^###^	6.9 ± 1.3^^*^##^
	LD-DD Phase angle (min)	-3.5 ± 32.4	-27.9 ± 15.4	32.3 ± 24.4	15.5 ± 24.9
**DD**	Period (hr)	23.8 ± 0.2	23.8 ± 0.1	23.8 ± 0.2	23.7 ± 0.1*^##^
	% V	53.0 ± 12.9	59.4 ± 6.7	27.8 ± 8.1^^^	29.9 ± 5.2^^^
	Activity (rev/hr)	872 ± 177	1040 ± 126^#^	474 ± 192^^	590 ± 205^^^^###^
	Alpha (min)	557 ± 23	619 ± 69*	612 ± 134^	690 ± 52^*
	Imprecision (min)	25.1 ± 6.1	13.5 ± 5.8***	23.1 ± 6.5^##^	23.2 ± 13.2^##^
	Fragmentation (bouts/day)	5.4 ± 1.1	4.0 ± 0.7	9.1 ± 0.8^^^^###^	7.5 ± 1.4^^^*^#^
	CT16 LP (min)	60.3 ± 35.9^###^	103 ± 19*	69.1 ± 10.2	90.4 ± 20.7^#^

When main or interaction effects were identified, significant genotypic differences within sex and age, sex differences within age and genotype, and age differences within sex and genotype were identified post-hoc using the Holm-Sidak method for multiple pairwise comparisons

^Genotype, P < 0.05;

^^Genotype, P < 0.01;

^^^Genotype, P < 0.001;

*Sex, P < 0.05;

**Sex, P < 0.01;

***Sex, P < 0.001;

^#^Age, P < 0.05;

^##^Age, P < 0.01;

^###^Age, P < 0.001

Reported values are means ± 95% CI.

**Table 2:** Statistical analysis of locomotor behavior. Three-way ANOVA results testing effects of sex and age on BACHD mouse locomotor activity rhythm parameters (DF = 63).

		Age		Genotype		Sex		Age x Genotype		Age x Sex		Genotype x Sex		Age x Genotype x Sex	
	Parameter	F	P	F	P	F	P	F	P	F	P	F	P	F	P
**LD**	% V	5.8	0.02	129	<0.001	7.33	0.009	13.9	<0.001	0.592	NS	0.005	NS	1.634	NS
	Activity (rev/hr)	14.3	<0.001	42.8	<0.001	20.7	<0.001	3.82	NS	4.2	0.05	1.17	NS	0.757	NS
	% Activity in Light	4.9	0.03	21.2	<0.001	0.929	NS	6.44	0.01	10.7	0.002	3.28	NS	1.623	NS
	Alpha (min)	1.93	NS	18.4	<0.001	5.63	0.02	4.41	0.04	4.22	0.05	6.82	0.01	2.438	NS
	Imprecision (min)	2.07	NS	38.4	<0.001	8.19	0.006	8	0.006	0.033	NS	8.42	0.005	4.757	0.03
	Fragmentation (bouts/day)	7.69	0.008	45.8	<0.001	12.1	<0.001	49.1	<0.001	0.757	NS	2.98	NS	1.53	NS
	LD-DD Phase angle (min)	3.02	NS	3.45	NS	10.2	0.002	6.74	0.01	0.772	NS	2.57	NS	1.38	NS
**DD**	Period (hr)	1.07	NS	2.34	NS	1.11	NS	4.44	0.04	0.56	NS	0.484	NS	3.31	NS
	% V	12.9	<0.001	57.4	<0.001	2.87	NS	5.93	0.02	0.018	NS	0.759	NS	0.01	NS
	Activity (rev/hr)	34.3	<0.001	38.9	<0.001	20.9	<0.001	3.41	NS	3.53	NS	1.9	NS	3.42	NS
	Alpha (min)	0.069	NS	5.31	0.03	5.69	0.02	0.197	NS	0.48	NS	0.245	NS	0.706	NS
	Imprecision (min)	5.59	0.02	0.242	NS	6.65	0.01	4.05	0.05	0.078	NS	5.94	0.02	0.008	NS
	Fragmentation (bouts/day)	14.7	<0.001	45	<0.001	4.95	0.03	14.9	<0.001	5.13	0.03	0.06	NS	0.007	NS
	CT16 LP (min)	18.7	<0.001	1.99	NS	18.1	<0.001	1.29	NS	0.227	NS	0.009	NS	2.81	0.1

To understand how disease progression develops in females, we compared BACHD female wheel-running activity rhythms to that of WT female littermate controls (S2 Fig; Tables 1 and 2). As in male BACHD (S3 Fig; Tables 1 and 2), BACHD female mice displayed reduced power rhythms in LD and DD relative to female controls. The rhythms in the female BACHD mice were more fragmented than WT females by 6 months. Unlike males, BACHD female activity levels were protected relative to WT females at 3 months of age. BACHD female activity levels did significantly decline relative to WT females by 6 months of age. Also unlike males, the precision of female rhythms was protected in BACHD females at both 3 and 6 months of age (Tables 1 and 2).

### Male and female SCN pathophysiology in BACHD mice

**Fig 2:**
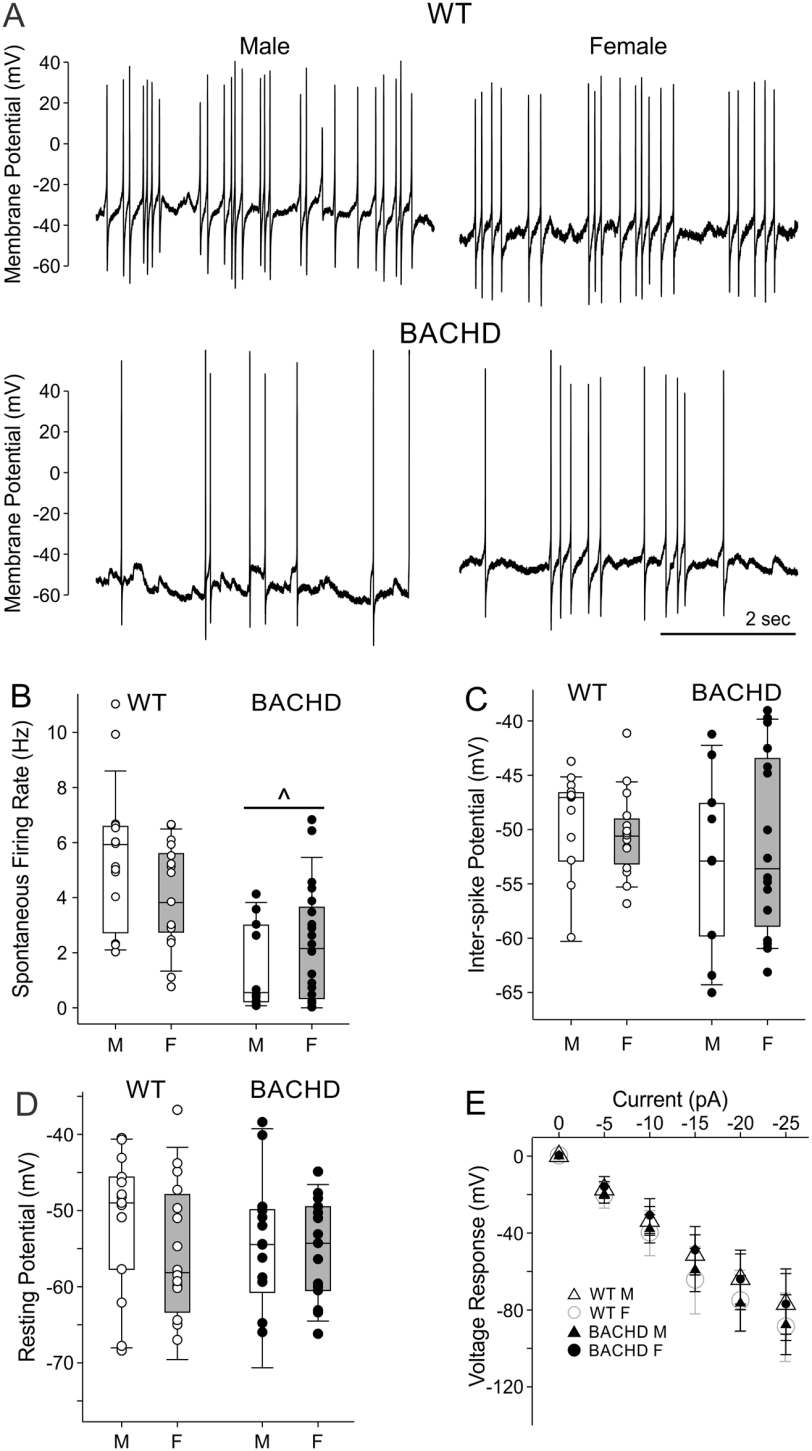
Daytime neural activity is comparably depressed in female and male BACHD SCN neurons. (A) Representative traces of SCN neuron spontaneous electrical activity recorded during the day. (B-E) Box plots representing the first and third quartile (box), group medians (middle line) and data range (whiskers), of male (white boxes), and female (grey boxes) SCN neuron electrophysiological properties, with data points superimposed for individual WT (white dots) and BACHD (black dots) neurons. Two-Way ANOVA was used to identify possible effects of genotype and sex while post-hoc multiple pairwise comparison testing was carried out with Holm-Sidak method (*P* < 0.05; Table 2). (B) BACHD SCN neuron SFR was reduced during the daytime relative to WT. (C) Inter-spike membrane potential was not altered by sex or genotype. (D) No sex or genotype differences in membrane potential recorded in the presence of TTX (1 μM) and gabazine (10 μM) to silence synaptic and electrical activity. (E) Baseline subtracted voltage responses are plotted for each group as means ± 95% CI’s. Two-Way ANOVA detected significant main effects of current injection, and an interaction of sex and genotype on voltage responses, but post-hoc pairwise comparisons using Two-Tailed T-Tests (*P* < 0.05) failed to detect significant voltage response differences for groups at any particular current injection magnitude. ^*P* < 0.05.

A fundamental feature of SCN neurons is that they are intrinsic pacemakers that generate spontaneous APs during the daytime. Possible genotype and sex differences in SFR, AP properties and resting membrane potential in male and female BACHD and WT mice were evaluated at 3 months of age (Fig 2; Tables 3 and 4). We found that daytime SFR was depressed in both BACHD male and BACHD female mouse SCN neurons relative to WT controls (Fig 2A and 2B). We did not find an impact of sex on SFR although this study may have been underpowered to detect a subtle sex difference in SCN pathophysiology. AP amplitude did show significant interaction effects of sex and genotype (Table 4) but no additional effects of sex and genotype were identified (Table 4). The inter-spike membrane potential did not vary with sex or genotype (Fig 2C). Finally, resting membrane properties were examined in electrically silent neurons using bath application of TTX (1 μM) to inhibit AP generation, and gabazine (10 μM) to silence synaptic activity. We did not find a difference in the resting membrane potential nor a difference the neurons voltage-response to current injection in BACHD SCN (Fig 2D and 2E; Tables 3 and 4). In summary, both male and female BACHD SCN neurons show evidence of depressed firing rate without any clear sex differences in this critical physiological measure.

**Table 3:** Genotype and sex effects on SCN neuron electrophysiological properties. Two-way ANOVA results testing effects of genotype and sex on daytime action potential and resting membrane properties in WT and BACHD SCN neurons (top). When main or interaction effects were identified, significant genotypic differences within sex, and significant sex differences within genotype, were identified post-hoc using the Holm-Sidak method for multiple pairwise comparisons.

	**WT**		**BACHD**	
**Action Potential Properties**	**Male (n = 19)**	**Female (n = 17)**	**Male (n = 10)**	**Female (n = 20)**
Spontaneous Firing Rate (Hz)	5.3 ± 1.3	4.0 ± 1.0	1.5 ± 1.1^^^	2.3 ± 1.0^^
Instantaneous Frequency (Hz)	4.8 ± 1.4	4.8 ± 1.0	2.1 ± 1.3^^	3.1 ± 1.4^
Interevent Interval (msec)	283 ± 127	230 ± 48	981 ± 889^^	696 ± 459
Inter-Spike Membrane Potential (mV)	-50.0 ± 3.3	-50.5 ± 2.2	-52.8 ± 5.7	-51.3 ± 4.5
Peak Amplitude (mV)	94.0 ± 12.5	112 ± 9.0*	114 ± 15.9^	95.5 ± 15.1*
Time to Peak (msec)	2.4 ± 0.2	2.2 ± 0.3	2.1 ± 0.3	2.1 ± 0.2
Half-Amplitude (mV)	47.2 ± 6.3	55.6 ± 4.5*	56.9 ± 7.8^	47.2 ± 8.0^*
Time to Rise Half Amplitude (msec)	0.74 ± 0.29	0.69 ± 0.22	0.58 ± 0.33	0.47 ± 0.24
Rise Tau (msec)	4.4 ± 1.8	5.9 ± 2.6	6.2 ± 4.3	4.8 ± 1.5
Area (mV*msec)	313 ± 42	354 ± 78	350 ± 84	306 ± 25
Half-Width (msec)	2.2 ± 0.6	2.2 ± 0.4	1.9 ± 0.6	2.1 ± 0.9
Antipeak Amplitude (mV)	9.4 ± 11.0	13.2 ± 12.0	21.1 ± 19.8	25.2 ± 8.7
Time to Antipeak (msec)	4.8 ± 0.3	4.1 ± 0.9	4.3 ± 0.7	3.8 ± 0.8
Time to Decay Half Amplitude (msec)	3.9 ± 0.4	3.7 ± 0.6	3.5 ± 0.9	3.4 ± 0.3
Decay Time (msec)	0.65 ± 0.33	0.59 ± 0.33	0.57 ± 0.43	0.25 ± 0.29
Decay Slope (mV/msec)	-32.9 ± 21.4	-40.7 ± 26.5	-41.9 ± 34.2	-10.5 ± 13.6
Decay Tau (msec)	16.1 ± 11.2	12.2 ± 12.0	17.9 ± 15.5	10.6 ± 7.2
**Resting Membrane Properties**	**Male (n = 16)**	**Female (n = 17)**	**Male (n = 17)**	**Female (n = 14)**
Capacitance (pF)	9.1 ± 1.4	9.0 ± 1.8	10.4 ± 2.1	10.7 ± 2.4
Resting Membrane Potential (mV)	-51.2 ± 4.9	-56.0 ± 5.3	-55.0 ± 5.6	-55.2 ± 3.7
Input Resistance (MΩ)	2.7 ± 0.1	3.2 ± 0.1*	3.1 ± 0.14^	2.6 ± 0.2^^*
Hyperpolarization Slope (mV/msec)	-1.3 ± 0.2	-1.8 ± 0.3**	-1.5 ± 0.2	-1.4 ± 0.2^
Hyperpolarization Area (mV*msec)	-13220 ± 2130	-15570 ± 2470**	-14520 ± 2210	-13710 ± 2480^
Hyperpolarization Peak Time (msec)	804 ± 29	771 ± 25	794 ± 27	774 ± 32

^Genotype within Sex, *P* < 0.05;

^^ Genotype within Sex, *P* < 0.01;

^^^ Genotype within Sex, *P* < 0.001;

*Sex within Genotype, *P* < 0.05;

**Sex within Genotype, *P* < 0.01.

Reported values are mean ± 95% CI (bottom).

**Table 4:** Statistical analysis of electrophysiological data. Two-way ANOVA results testing effects of genotype and sex on daytime action potential and resting membrane properties in WT and BACHD SCN neurons.

		**Genotype**		**Sex**		**Genotype X Sex**	
**Action Potential Properties**	DF	F	P	F	P	F	P
Spontaneous Firing Rate (Hz)	65	24	<0.001	0.183	0.67	3.325	0.073
Instantaneous Frequency (Hz)	47	14.4	<0.001	0.827	0.368	0.695	0.409
Interevent Interval (msec)	49	10.194	0.003	0.858	0.359	0.406	0.527
Inter-Spike Membrane Potential (mV)	54	1.008	0.32	0.073	0.788	0.304	0.584
Peak Amplitude (mV)	50	0.104	0.749	0.005	0.942	8.776	0.005
Time to Peak (msec)	50	4.159	0.047	1.337	0.253	0.52	0.475
Half-Amplitude (mV)	49	0.042	0.839	0.034	0.854	9.215	0.004
Time to Rise Half Amplitude (msec)	50	2.284	0.137	0.406	0.527	0.067	0.794
Rise Tau (msec)	50	0.117	0.734	0.002	0.967	1.495	0.228
Area (mV*msec)	49	0.401	0.53	2.404	0.128	0.377	0.542
Half-Width (msec)	50	1.674	0.202	0.57	0.454	0.092	0.763
Time to Decay Half Amplitude (msec)	47	1.903	0.175	0.537	0.468	0.115	0.763
Decay Time (msec)	49	1.575	0.216	2.59	0.114	0.297	0.588
Decay Slope (mV/msec)	49	0.854	0.36	0.709	0.404	0.269	0.606
Decay Tau (msec)	49	0.401	0.53	2.404	0.128	0.377	0.542
**Resting Membrane Properties**							
Capacitance (pF)	55	2.823	0.099	0.016	0.901	0.076	0.784
Resting Membrane Potential (mV)	59	0.431	0.514	1.163	0.285	0.979	0.327
Input Resistance (mΩ)	657	0.252	0.616	0.021	0.884	11.628	<0.001
Hyperpolarization Slope (mV/msec)	343	0.887	0.347	2.31	0.13	4.559	0.033
Hyperpolarization Area (mV/msec)	353	0.053	0.818	3.422	0.065	0.206	0.65

### Anatomical abnormalities in the SCN of male BACHD mice

**Fig 3:**
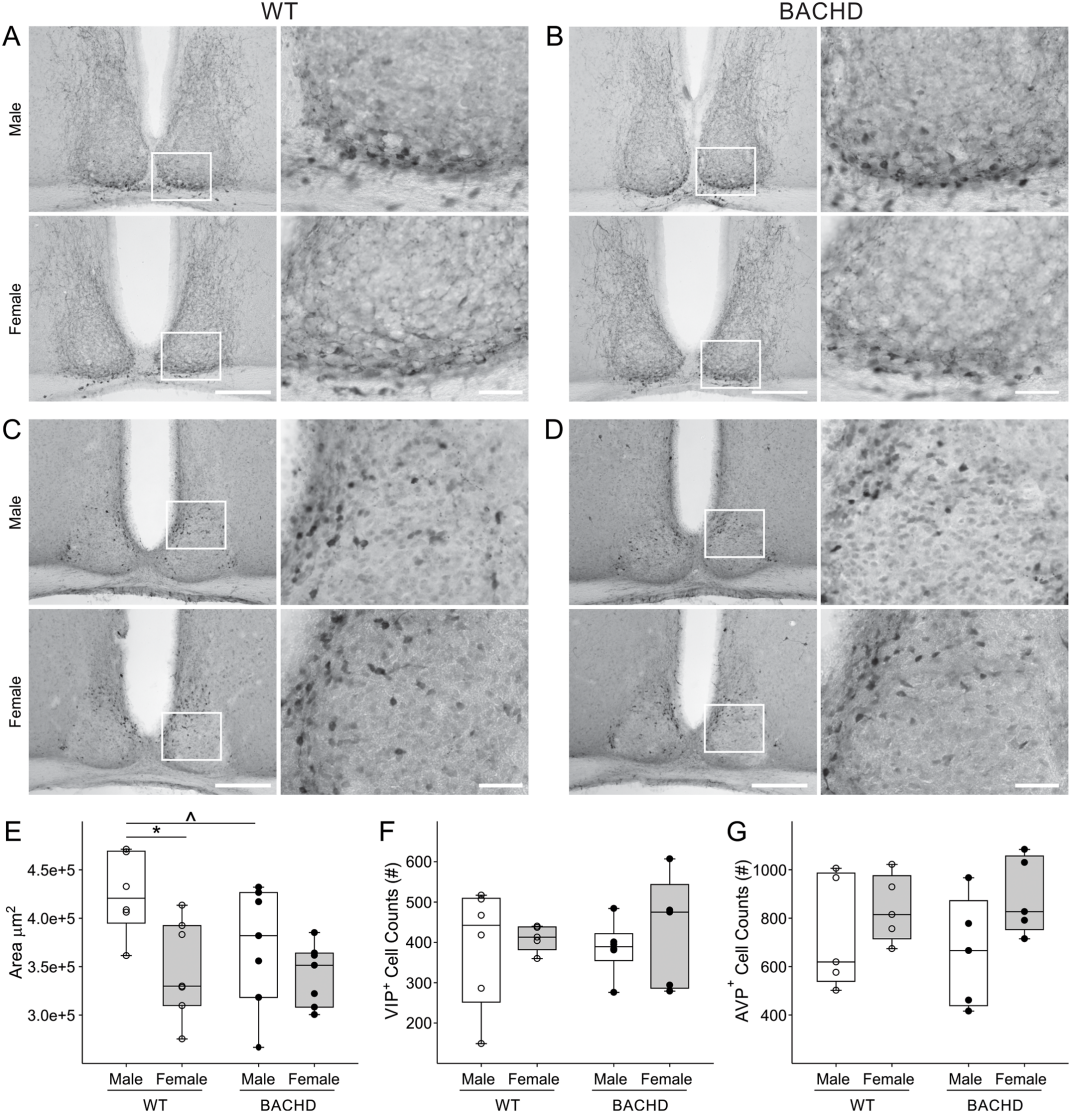
Sex and genotypic difference in the size of the SCN of WT and BACHD mice. Representative images of the SCN, stained with VIP (A & B) or AVP (C &D) and counterstained with Nissl. Boxed regions in 10x image (left, scale bar = 200μm) are magnified at 40x (right, scale bar = 40μm). (E) Measurements of the Nissl-defined SCN revealed a significantly smaller SCN in 3 month-old WT females compared to age-matched WT male mice. WT females displayed a smaller and elongated SCN, while WT male SCN was larger and round. A significant reduction in the area of the SCN was found in BACHD males as compared to age-matched WT males, while females BACHD did not display pathology-associated variations. Individual data points represent the average of the left and right area of the SCN of each animal (n = 6–7) measured by 2 observers masked to the sex and genotype. No sex or genotypic differences were found in the number of VIP (F) and AVP (G) positive neurons. Individual data points represent the number of positive cell counted per animal for each group (n = 4–6). Individual data points are superimposed onto box plots representing first and third quartile (box), group medians (middle line) and data range (whiskers) for each group. Main effects of sex and genotype were identified by Two-way ANOVA *P<0.05 (see also Table 6). Significant genotypic differences within sex were identified post-hoc by Two-Tailed T-Tests, with ^*P* < 0.05.

The SCN is the brain region responsible for controlling circadian rhythms and the temporal patterning of sleep. To determine if there were sex differences in the SCN structure, we measured the Nissl-defined SCN as well as counted the number of VIP and AVP expressing neurons in male and female BACHD and WT mice at 3 months of age (Fig 3; Table 5). We found that sex influenced the size and shape of the SCN, with WT females displaying a significantly smaller (18%) and narrower (10%) SCN (Tables 5 and 6). No differences were found in the SCN size or shape between WT and BACHD females (Fig 3; Table 5). Conversely, the area of the SCN in BACHD males was significantly reduced by about 13% as compared to WT (Fig 3; Tables 5 and 6). The height of the dorsal-ventral axis was significantly decreased by 11% (Fig 3; Tables 5 and 6) in mutant mice, whilst the width was unchanged. We did not find a significant effect of sex or genotype on the number of VIP or AVP expressing neurons (Fig 3; Tables 5 and 6). These findings suggest that the HD mutation selectively influences the SCN structures in males without altering AVP and VIP expression in early adulthood.

**Table 5:** Genotype and sex differences in BACHD SCN anatomy. Effects of sex and genotype on different histological parameters of the SCN and the number of VIP and AVP neurons. When main or interaction effects were identified, significant sex differences within genotype, and genotype differences within sex, were identified post-hoc using the Two-Tailed T-Tests, with P < 0.05.

	**WT**		**BACHD**	
	**Male (n = 6)**	**Female (n = 7)**	**Male (n = 7)**	**Female (n = 7)**
Area (μm^2^)	424901 ± 17082	347586 ± 18859*	371253 ± 23390^	341905 ± 12056
Height (μm)	2132 ± 58	1920.7 ± 75	1912.3 ± 95^	1939.9 ± 54
Width (μm)	1970 ± 98	1743 ± 39*	1878 ± 88	1744 ± 27
	**Males (n = 5–6)**	**Females (n = 5)**	**Males (n = 5–6)**	**Females (n = 5)**
VIP^+^ cells	391 ± 152	411 ± 40	387 ± 70	427 ± 172
AVP^+^ cells	734 ± 291	839 ± 171	658 ± 283	889 ± 198

*Sex within genotype, P < 0.05;

^Genotype within sex, P < 0.05.

Mean ± SEM of the average of the left and right area, height and width of the SCN of male and female mice of each genotype. For the VIP and AVP cell number reported values are mean ± 95% CI. Two-Tailed T-Tests

**Table 6:** Statistical analysis of anatomical data. Two-way ANOVA results testing effects of sex and genotype on different histological parameters of the SCN and the number of VIP and AVP positive cells.

		Genotype		Sex		Genotype x Sex	
	DF	F	P	F	P	F	P
Area (μm^2^)	23	2.577	NS	8.33	0.0083	1.685	NS
Height (μm)	23	1.853	NS	1.556	NS	2.634	NS
Width (μm)	23	0.453	NS	7.021	0.0143	0.467	NS
VIP^+^ cells	18	0.02	NS	0.43	NS	0.05	NS
AVP^+^ cells	16	0.023	NS	3.75	NS	0.53	NS

### Sex differences in motor coordination and body weight

**Fig 4:**
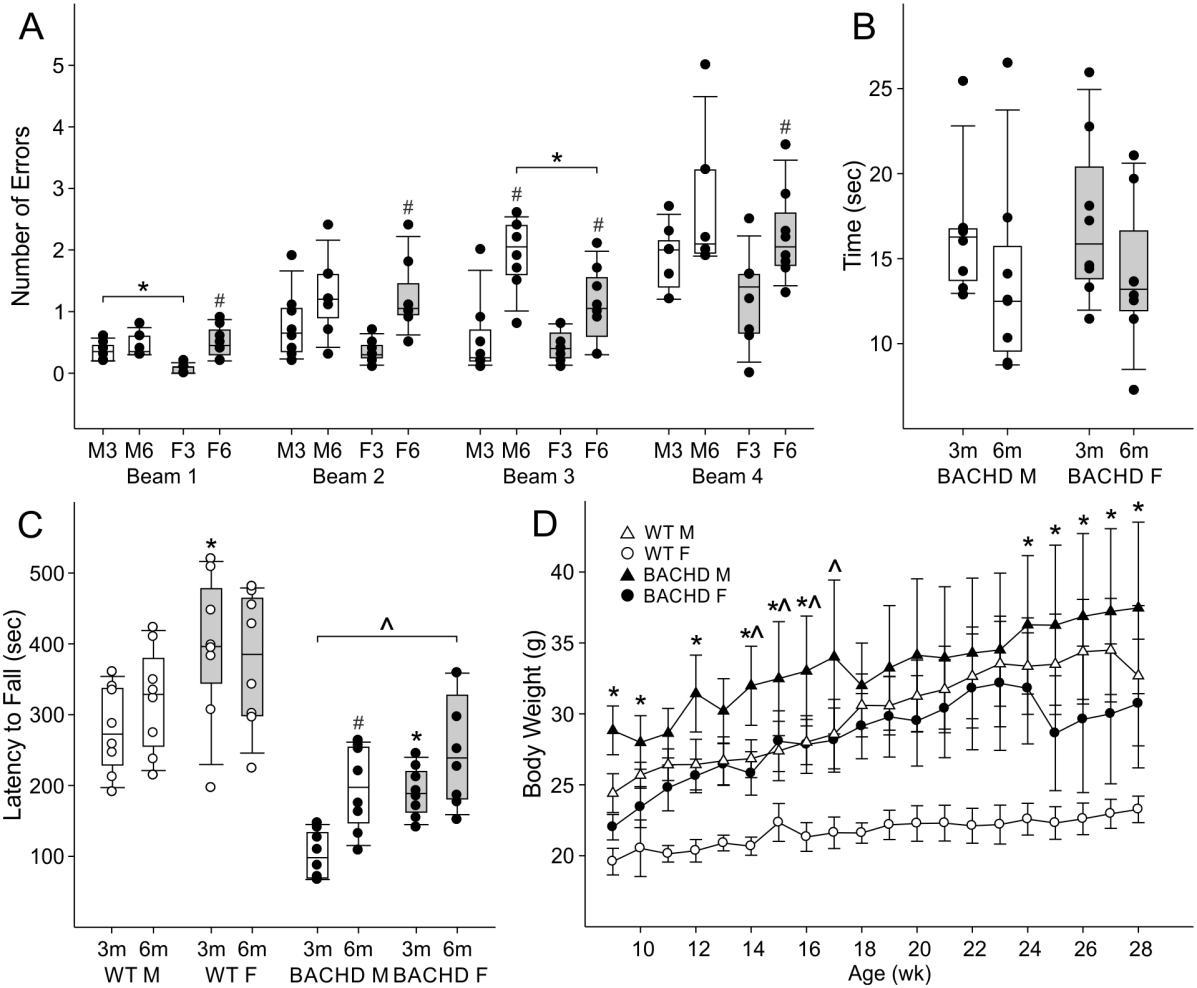
BACHD mouse sex differences in motor coordination and body weight. (A-C) Box plots represent first and third quartile (box), group medians (middle line) and data range (whiskers), of male (white boxes) and female (grey boxes) mice, with individual data points superimposed. (A) BACHD mouse step errors on progressively narrowing challenging beams (Beams 1–4) for males at 3 months (M3) and 6 months (M6), as well as females at 3 months (F3) and 6 months (F6). Two-Way ANOVA identified main effects of sex and age on step errors. Significant sex differences within age (*) and/or significant age differences within sex (#) for each beam were identified post-hoc, using Holm-Sidak method for multiple pairwise comparisons, with *P* < 0.05. (B) Time to traverse all 4 challenging beams. (C) Rotorod latency to fall for WT (white dots) and BACHD (black dots) mice. Three-Way ANOVA identified main effects of genotype, and interactions of sex, age, and genotype on latency to fall. Significant genotypic differences within age and sex (^), sex differences within genotype and age (*), and significant age differences within sex and genotype (#), on latency to fall were identified post-hoc, using Two-Tailed T-Tests, with *P* < 0.05. (D) Body weight of WT and BACHD mice. Three-Way ANOVA identified main effects of genotype, sex, and age as well as the interaction of genotype and sex as well as sex and age (Table 4) on body weight. Post-hoc pairwise comparisons using Holm-Sidak method identified that at all age points, WT females weighed less than WT males and BACHD females, as well as ages significant differences in weight were detected between BACHD male and female mice (*P < 0.05), or BACHD male and WT male mice (^*P* < 0.05). Points represent mean, and error bars 95% CI.

Finally, we measured motor performance (challenge beam and rotarod tests) in male and female BACHD and WT mice at 3 months and 6 months of age (Fig 4; Tables 7 and 8). With the challenge beam, all BACHD mice made more step errors on the narrower beams than the wider beams at both 3 and 6 months of age (Fig 4A; Table 7). We observed sex differences in step error numbers at both ages. At 3 months of age, BACHD females made significantly fewer errors on the first and widest beam. Age-related deterioration in motor control largely occurred only for females. By 6 months, BACHD female step errors were on par with male’s except for on the third beam, for which BACHD males still performed significantly worse than females. Interestingly, this is also the only beam on which males made more step errors between 3 and 6 months of age. We also examined the time it took the animals to cross the challenge beam, but neither sex nor age had a statistically significant effect (Fig 4B; Table 7).

**Table 7:** Sex differences in BACHD motor coordination deterioration. Two-way ANOVA results testing effects of sex and age on challenge beam step errors. When main or interaction effects were identified, significant sex differences within age, and age differences within sex, were identified post-hoc using the Holm-Sidak method for multiple pairwise comparisons.

**3 months**	**Parameter**	**Males (n = 8)**	**Females (n = 8)**		**6 months**	**Parameter**	**Males (n = 8)**	**Females (n = 8)**
**Challenge Beam**	Beam 1	0.3 ± 0.1	0.1 ± 0.1*		**Challenge Beam**	Beam 1	0.4 ± 0.1	0.5 ± 0.2^##^
	Beam 2	0.8 ± 0.5	0.3 ± 0.2			Beam 2	1.36 ± 0.5	1.2 ± 0.5^##^
	Beam 3	0.5 ± 0.5	0.4 ± 0.2			Beam 3	1.9 ± 0.5^#^	1.1 ± 0.5^#^*
	Beam 4	1.9 ± 0.4	1.2 ± 0.6			Beam 4	2.7 ± 0.9	2.2 ± 0.6^#^
	Time	16.5 ± 3.3	17.2 ± 4.1			Time	13.8 ± 4.9	14.0 ± 3.7
			**Sex Effect**		**Age Effect**		**Interaction**	
	**Parameter**	DF	F	P	F	P	F	P
**Challenge Beam**	Beam 1	31	3.10	NS	16.87	<0.001	0.117	0.02
	Beam 2		1.56	NS	13.56	<0.001	4.22	NS
	Beam 3		5.89	0.022	27.4	<0.001	3.43	NS
	Beam 4		3.79	NS	9.98	0.004	0.09	NS
	Time		0.06	NS	2.9	NS	0.03	NS

*Sex within Genotype, *P* < 0.05;

^#^Age within Sex, *P* < 0.01;

^##^Age within Sex, *P* < 0.01.

Reported values are mean ± 95% CI

**Table 8:** Sex differences in BACHD motor coordination deterioration. Table shows the latencies to fall off rotarod (sec).

	WT		BACHD		
Rotorod	Male (n = 8)	Female (n = 8)	Male (n = 8)	Female (n = 8)
3 months	279 ± 52	394 ± 89**	196 ± 50^	191 ± 30^^
6 months	321 ± 63	375 ± 81	102 ± 28 ^^^#^	251 ± 67^***

**Sex within genotype, P < 0.01;

***Sex within genotype, P < 0.001;

^Genotype within sex, P < 0.05;

^^Genotype within sex, P < 0.01.

Reported values are mean ± 95% CI.

We also tested motor coordination using the rotarod test and found a significant interaction of genotype, sex, and age on performance (Fig 4C; Tables 8 and 9). First, compared to WT controls, BACHD rotarod latencies to fall were shorter at both 3 months and 6 months of age. Previous studies did not find sex differences among BACHD mouse performance using this test (34). Similarly, at 3 months, we did not observe sex differences in the latencies to fall off rotarod for BACHD mice. However, we found that while BACHD male mice exhibited age related decline in rotarod performance, BACHD females did not, so that by 6 months BACHD female rotarod performance was significantly better than that of BACHD males but still poor compared to WT females. We also found that WT female littermates performed significantly better than WT males, but only at 3 months of age. This sex difference did not persist at 6 months of age. In summary, challenge beam and rotarod performance showed age-related impairment for BACHD mice that occurred later for females.

**Table 9:** Statistical analysis of Sex differences in BACHD motor coordination deterioration and body weight. Three-way ANOVA results testing effects of genotype, sex, and age on rotorod latency to fall and body weight. When main or interaction effects were identified, significant genotypic differences within sex, sex differences within genotype, as well as age differences within sex and genotype were identified post-hoc using the Two-Tailed T-Tests, with P < 0.05.

	Genotype x sex		Genotype x Age		Sex x Age		Genotype x Sex x Age	
	F	P	F	P	F	P	F	P
**Rotorod**	0.13	0.72	0.6	0.4	1.62	0.2	8.6	0.005
**Body Weight**	18.1	<0.001	0.82	NS	2.54	<0.001	1.31	NS

Body weights of male and female BACHD and WT control animals was measured weekly throughout the study **(**Fig 4D; Table 8). WT males weighed more than WT females throughout early adulthood, and this difference in weight was greater at 6 months when there was no sex difference in motor coordination, than at 3 months when WT females performed better on the rotarod. In contrast, BACHD mice variably show sex differences in body weight during the first 6 months of life (Fig 4). BACHD males weighed more on average than BACHD females until 15 weeks, but then BACHD females rapidly gained weight from 18–23 weeks and had similar weight to BACHD males. This rapid weight gain ended for BACHD females before BACHD males stopped growing, so that after 24 weeks BACHD males were again significantly heavier than BACHD females, albeit to a smaller degree than the sex difference in bodyweight observed in WT mice. Nevertheless, although BACHD and WT females weighed less than their male counterparts did at both ages, they did not consistently perform better on motor challenges. Sex differences in motor performance occurred among WT animals only at 3 months of age, and for BACHD animals only at 6 months of age. Thus, genotypic differences in weight does not account for deficits in rotarod performance observed in BACHD animals. BACHD males weighed significantly more than WT males only until 4 months of age, and BACHD females weighed as much as WT males from 2 to 6 months of age, but both BACHD sexes consistently performed worse on rotarod relative to WT males. Taken together, these findings suggest that body weight cannot account for the impaired motor coordination we observed in BACHD mice, nor the sex difference in rotarod performance of young WT mice or aged BACHD mice.

## Discussion

Sex differences in circadian behavior have been seen in many animals [43, 48–54]. In mice, SCN-regulated behavioral patterning characteristics, such as activity onset, the closely-related free running activity rhythm period, activity offsets, activity rates, bouts and durations, sleep onsets, as well as light-induced behavioral rhythm phase shift magnitudes, are regulated by gonadal hormones and/or sex chromosomes [43, 55–61]. The fact that many of these parameters also show sex differences in humans [62–67], and are altered in individuals with HD [8,24,30,32,68], led us to investigate whether there are sex differences in circadian system dysfunction in a mouse carrying the human HD gene. We focused our investigation on the period of time (3–6 months) during which the greatest decline in behavioral and physiological output rhythms occurs in male BACHD mice [36], and found that at the level of behavioral output, BACHD male mice have more severe circadian system dysfunction than BACHD females (Fig 1). By 6 months, BACHD male activity onset times were more variable, their activity bouts were more fragmented, and they were less nocturnal than BACHD females. Notably, the deterioration of the male circadian system was also associated with more severe motor dysfunction (Fig 4).

The ability to align behavior to a predictably rhythmic environment is one of the key functions of the circadian timing system and, for humans, it is conducive to maintaining a regular work/life schedule [69, 70]. Increased variability in activity onset times in LD (but not DD), and increased activity during the light indicate that BACHD males have more severe deficits in their circadian system’s ability to entrain to a rhythmic photic environment. These deficits are also evidenced by the increased number of days the BACHD males require to align behavioral rhythms to acute phase shifts of their environmental LD cycle relative to WT male controls [36].

Depressed daytime SCN neuron firing rates (Fig 2) were associated with a significant reduction in behavioral rhythm power for both BACHD sexes (Tables 1 and 2). Part of the natural aging process results in reduced behavioral and physiological rhythm power, and increased behavioral fragmentation [71,72]. In aged WT mice these phenotypes are associated with reduced sleep efficiency and reduced amplitude electrical output from SCN caused by the dysregulation of potassium currents active during the inter-spike interval [72]. BACHD mice showed a similar loss in SCN electrical activity rhythms due to depressed daytime SCN electrical activity, but neither BACHD male or female SCN neurons show daytime membrane potential hyperpolarization (Fig 2C and 2D), suggesting that like aged SCN, currents regulating inter-spike intervals rather than membrane potential may underlie their pathophysiology. Even though aged WT and BACHD mouse SCNs lose rhythms in electrical excitability, they maintain rhythms in clock gene expression [36, 71], suggesting the mechanism disrupting rhythmic physiology of aged and BACHD mouse SCN neurons is post-translational in nature.

Sexual dimorphism in the shape and cell number of the SCN has been previously reported in humans and other species [73–77]. To our knowledge, the present study provides the first evidence that these sex differences in the size and shape of the SCN also exist in the mouse brain (Fig 3). Here we report that female SCN appears to have an elongated shape while in males, these nuclei are more spherical which broadly mirrors the differences in humans [73]. In addition, we found that the BACHD males had a significantly smaller and shorter SCN as compared to WT males. In contrast, no differences in the area, height or width of the SCN were found between females WT and BACHD. Additionally, even though we did not find evidence for reduced AVP and VIP immunoreactivity in the SCN of the BACHD animals at 3 months of age (Fig 3), VIP has been reported to be reduced in the SCN of the R6/2 model of HD late in disease progress [78] as well as in HD patients post-mortem [79]. Our findings indicate that HD can selectively alter SCN architecture in males in early adulthood or, to put it another way, females may enjoy some projection against the structural changes driven by HD.

Why these sex differences emerge in the BACHD model is not clear. HTT is widely expressed in the central nervous system, including the SCN [80]. In the BACHD mouse model an estimated 5 copies of the mHTT transgene are inserted into the genome [33], but because the location of these insertion sites is not currently known we can only speculate whether there are sex differences in mHTT expression. If insertion sites are on sex chromosomes, sex differences in gene dosing related to sex differences in sex-chromosome compliment might account for sex differences in disease progression. More likely, the insertion sites are on autosomes like native HTT. The effects of mHtt on the ovary have not yet been characterized, but a mHtt-driven disruption in estrous cycling would be unlikely to account for the observed sex differences in behavioral parameters. Unlike other rodent models (rats and hamsters) that express estrus-phase-dependent changes in activity onsets [81, 82], female mice do not [43, 83]. Although some strains of female mice exhibit estrous cycle phase-dependent activity duration [83], we are unable to detect estrous-related-ultradian rhythms in activity duration or levels in our cohort of female mice [43]. On the other hand, young gonadectomized WT male mice display many of the same phenotypes as BACHD male mice, including greater reductions in activity levels, rhythm amplitude, and rhythm precision in LD relative to gonadectomized females [36, 43, 55, 84]. Although BACHD male mouse testosterone levels are unknown, HD patients and other mouse models of HD exhibit reduced testosterone levels [85–88]. Therefore, reduced circulating gonadal hormones could mediate some of the behavioral disruption in the BACHD model.

The paucity of clear evidence for sex difference in the human HD phenotype raises questions to the relevance of our mouse model findings for the human disease. Considering that HD is typically diagnosed at the end of a woman’s child bearing years, circa-menopause when estrogen levels drop severely [89], it may not be surprising that robust sex differences have not been observed in humans if they are mediated by estrogens. In addition, prior epidemiological studies have focused on tracking motor symptoms and may have missed sex differences in non-motor symptoms. Based on this study’s findings, we believe that an examination of sex differences in prodromal non-motor phenotypes is warranted, and considering alteration to melatonin rhythms are amongst the earliest disease phenotypes exhibited by HD gene carriers [32], it may be a worthwhile effort toward identifying early biomarkers of disease.

## Conclusions

Dysfunction in the circadian regulation of biological rhythms occurs early in the progression of HD and likely has negative health consequences that feed into the disease mechanism. Whether there are sex differences in sleep disruptions of HD gene carriers is unknown, but the observations outlined in this report are promising evidence that sex-specific factors mitigate some aspects of circadian dysfunction in HD. Furthermore, the less severe deficits in many activity rhythm parameters and motor coordination observed in BACHD females provide valuable evidence in support of the need to balance for sex in clinical studies.

## Supporting Information

S1 Fig Response to light exposure at CT16. (A-D) Representative double-plotted actograms of BACHD male, BACHD female, WT male, and WT female mouse circadian wheel-running activity rhythm phase shifts, in response to a 10 minute LP (100 lux) presented at CT16. The phase shift magnitude was determined by measuring the phase difference between best-fit regression lines (in red) drawn through the 10 days preceding, and 10 days subsequent to the LP treatment. Data collected at 3 months of age.
(TIF)

S2 Fig Female daily and circadian wheel running rhythms. (A-D) Representative double-plotted actograms of WT and BACHD female wheel running activity during 10 days in 12:12 LD (300 lux) and DD, at 3 and 6 months of age.
(TIF)

S3 Fig Male daily and circadian wheel running rhythms. (A-D) Representative double-plotted actograms of WT and BACHD male wheel running activity during 10 days in 12:12 LD (300 lux) and DD, at 3 and 6 months of age.
(TIF)

S1 Table Sex differences in BACHD SCN anatomy. Mean ± SEM of area, height and width of the left and right SCN of male and female mice of each genotype.
(TIFF)
