# Supplementary figures and images for: Sex Differences in Circadian Dysfunction in the BACHD Mouse Model of Huntington’s Disease
Source: PLoS One. 2016 Feb 12;11(2):e0147583. doi: 10.1371/journal.pone.0147583 (PMC4752447; doi:10.1371/journal.pone.0147583)

**A** 3mo BACHD male

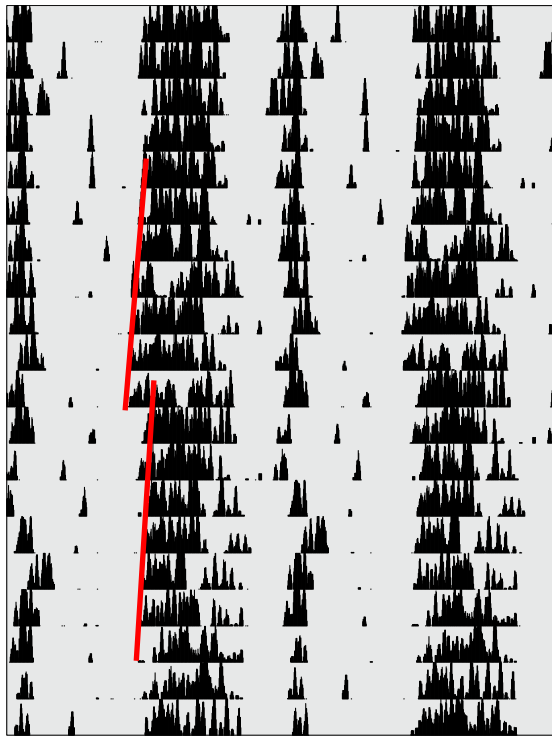

**B** 3mo BACHD female

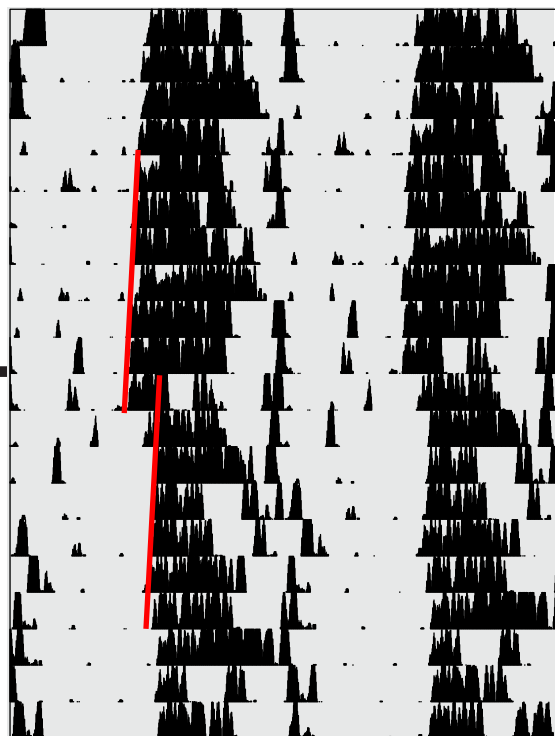

**C** 3mo WT male

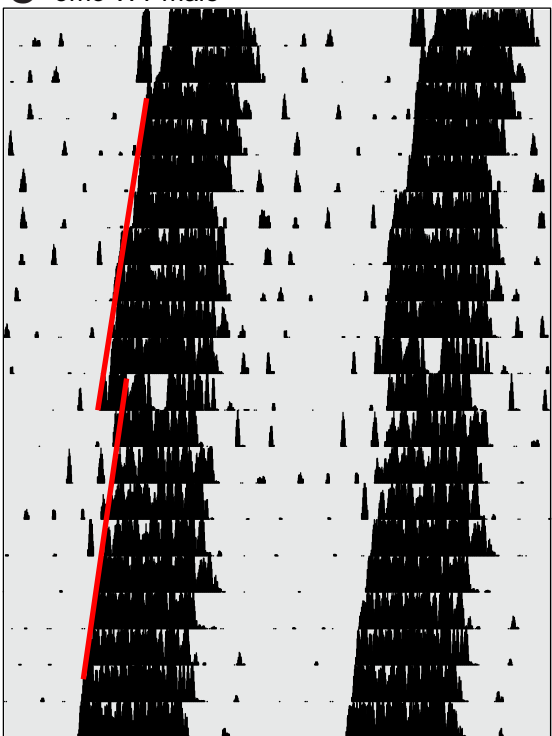

**D** 3mo WT female

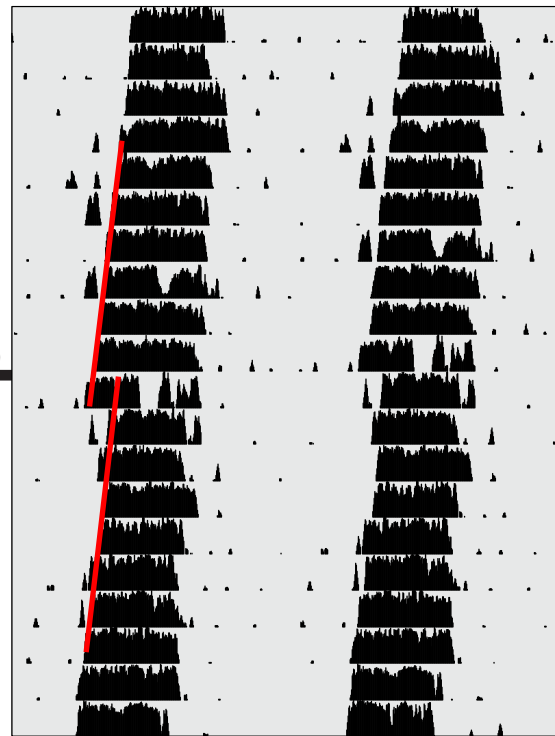

Supplement: S1 Fig — (A-D) Representative double-plotted actograms of BACHD male, BACHD female, WT male, and WT female mouse circadian wheel-running activity rhythm phase shifts, in response to a 10 minute LP (100 lux) presented at CT16. The phase shift magnitude was determined by measuring the phase difference between best-fit regression lines (in red) drawn through the 10 days preceding, and 10 days subsequent to the LP treatment. Data collected at 3 months of age. (TIF) [file pone.0147583.s001.tif]

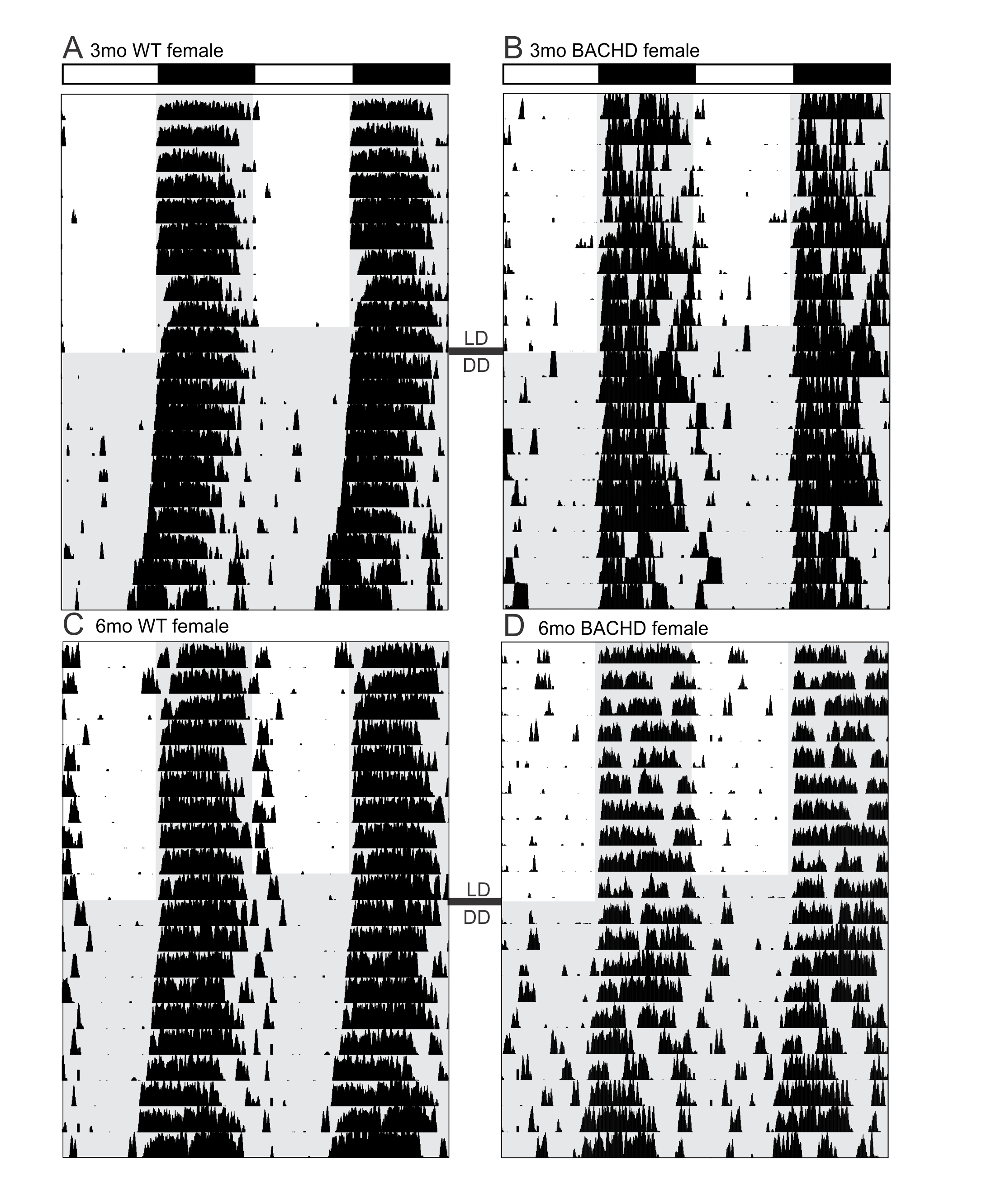

Supplement: S2 Fig — (A-D) Representative double-plotted actograms of WT and BACHD female wheel running activity during 10 days in 12:12 LD (300 lux) and DD, at 3 and 6 months of age. (TIF) [file pone.0147583.s002.tif]

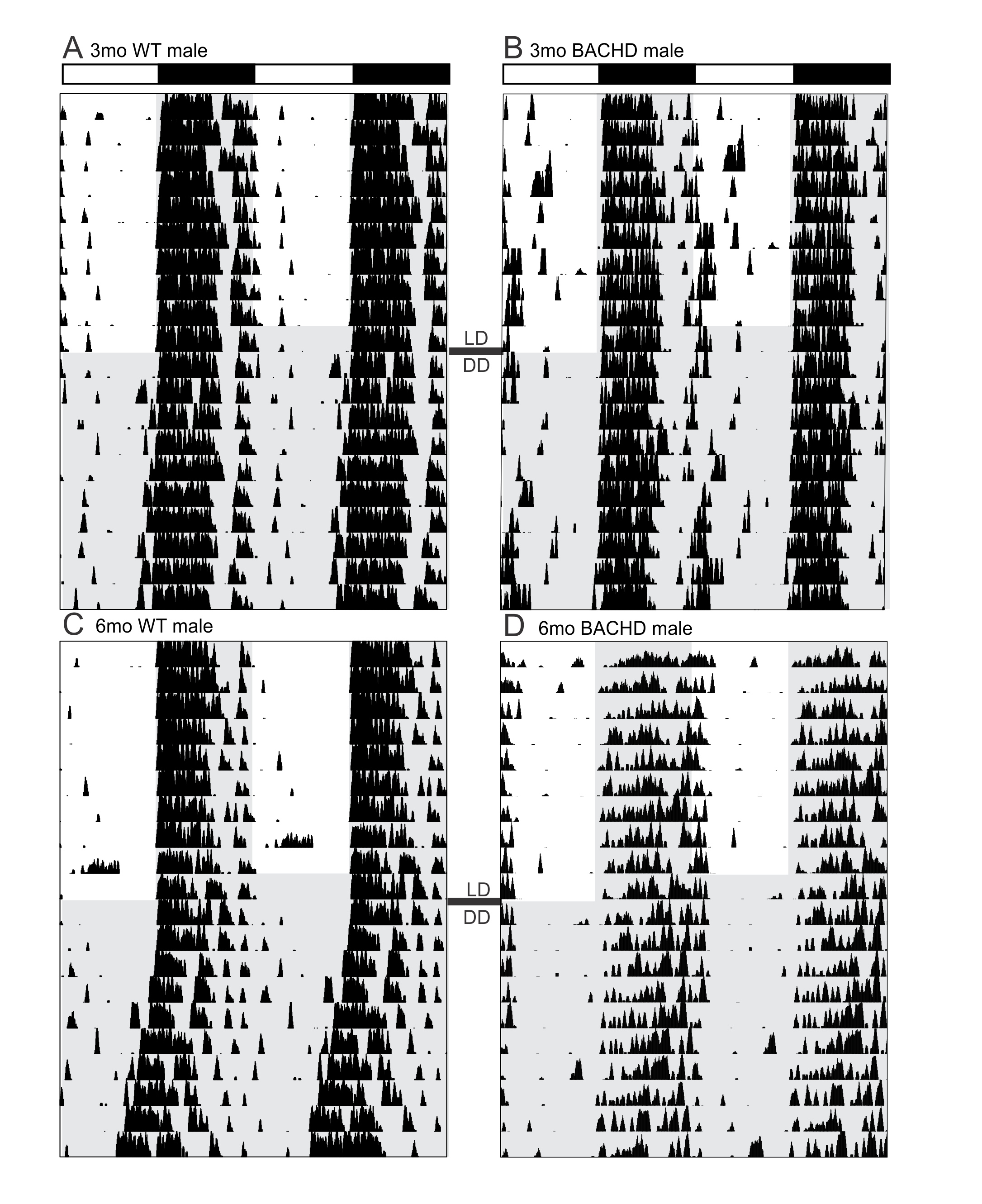

Supplement: S3 Fig — (A-D) Representative double-plotted actograms of WT and BACHD male wheel running activity during 10 days in 12:12 LD (300 lux) and DD, at 3 and 6 months of age. (TIF) [file pone.0147583.s003.tif]
